# How to avoid harm with feeding critically ill patients: a synthesis of viewpoints of a basic scientist, dietitian and intensivist

**DOI:** 10.1186/s13054-023-04543-1

**Published:** 2023-07-01

**Authors:** Annika Reintam Blaser, Olav Rooyackers, Danielle E. Bear

**Affiliations:** 1 Department of Anaesthesiology and Intensive Care, University of Tartu, Puusepa 8, 50406 Tartu, Estonia; 2 Department of Intensive Care Medicine, Lucerne Cantonal Hospital, Lucerne, Switzerland; 3 Division of Anesthesiology and Intensive Care, Department of Clinical Science, Technology and Intervention, Karolinska Institutet, Huddinge, Sweden; 4 Department of Nutrition and Dietetics, Guy’s and St Thomas’ NHS Foundation Trust, London, UK; 5 Department of Critical Care, Guy’s and St Thomas’ NHS Foundation Trust, London, UK

**Keywords:** Nutrition, Enteral, Parenteral, Protein, Critically ill, Energy target

## Abstract

The optimal feeding strategy in critically ill patients is a matter of debate, with current guidelines recommending different strategies regarding energy and protein targets. Several recent trials have added to the debate and question our previous understanding of the provision of nutrition during critical illness. This narrative review aims to provide a summary of interpretation of recent evidence from the view of basic scientist, critical care dietitian and intensivist, resulting in joined suggestions for both clinical practice and future research. In the most recent randomised controlled trial (RCT), patients receiving 6 versus 25 kcal/kg/day by any route achieved readiness for ICU discharge earlier and had fewer GI complications. A second showed that high protein dosage may be harmful in patients with baseline acute kidney injury and more severe illness. Lastly, a prospective observational study using propensity score matched analysis suggested that early full feeding, especially enteral, compared to delayed feeding is associated with a higher 28-day mortality. Viewpoints from all three professionals point to the agreement that early full feeding is likely harmful, whereas important questions regarding the mechanisms of harm as well as on timing and optimal dose of nutrition for individual patients remain unanswered and warrant future studies. For now, we suggest giving low dose of energy and protein during the first few days in the ICU and apply individualised approach based on assumed metabolic state according to the trajectory of illness thereafter. At the same time, we encourage research to develop better tools to monitor metabolism and the nutritional needs for the individual patient accurately and continuously.

## Background

Nutrition in critical illness has gained a lot of attention during the last few decades. It was previously suggested that early adequate feeding may improve outcomes and that “adequate” meant reaching full energy target early during critical illness. However, this concept was questioned following the publication of the EPaNIC trial in 2011 [1]. The worse outcomes in patients with early supplemental parenteral nutrition (PN) in this trial were first interpreted as being caused by the PN itself. This interpretation led to an abrupt reduction in PN in clinical practice [2] and recommendations against PN in guidelines [3]. Since then, there is cumulative evidence indicating that provision of full energy target (administering nutrition to cover full estimated energy expenditure) is harmful rather than beneficial independent of the route of delivery. This means that we are either overestimating the patient’s energy needs and are overfeeding or there is a biological benefit associated with underfeeding. Several studies have assessed possible biological mechanisms to explain this a priori unexpected finding. Mechanisms such as increased endogenous energy supply independent of exogenous energy provision and suppression of autophagy with nutrition have been studied and newly interpreted [4–6]. Several large clinical studies and meta-analyses addressing nutrition via different routes and/or in different doses have been published over recent years which question our previous understanding of the provision of nutrition during early critical illness [7–10].

In this narrative review, we summarise the findings of the most recent studies on the route and dose of nutrition as well as studies on explanatory mechanisms, providing interpretation of available evidence from the viewpoint of basic scientist, dietitian and intensivist, and offer joined suggestions for clinical practice and future research.

## Summary of recent studies

Both the CALORIES trial and NUTRIREA-2 trial, RCTs published several years ago, showed no difference in mortality when enteral nutrition (EN) and parenteral nutrition (PN) are used in similar doses. However, the latter RCT suggested possible harm from full EN in patients with shock when compared to full PN [7, 8]. That patients with full EN have more gastrointestinal (GI) symptoms as compared to PN is an expected finding; however, serious complications such as Ogilvie’s syndrome and acute mesenteric ischaemia being associated with EN was a finding requiring attention, even though the occurrence was rare [7, 11]. This finding was supported by a recently published (although conducted in 2015) propensity score matched prospective observational study (FRANS). The results showed that early full nutrition resulted in higher 28-day mortality vs. delayed nutrition with the effect attributed to early full EN rather than early PN [12]. Additionally, protein above 0.3 g/kg/day given in the first 48 h was associated with mortality in a dose-dependent manner. Importantly, the delayed nutrition group in this study, despite ‘not being fed’, received on average about 5 kcal/kg/day from non-nutritional sources (glucose and propofol) during the first 48 h. This is comparable to trophic feeding and only slightly lower than permissive underfeeding assessed in earlier studies [13, 14].

NUTRIREA-3, the most recent RCT in mechanically ventilated patients receiving vasopressors, confirmed the hypothesis that early full nutrition by any route is not beneficial but rather harmful [15]. This trial showed that aiming for low energy and protein (6 kcal/kg/day and 0.2–0.4 g/kg/day) versus full targets (25 kcal/kg/day and 1.0–1.3 g/kg/day, respectively) by any route during the first week in the ICU was associated with shorter time to ‘readiness to discharge’ and less complications. Reduced complications included vomiting, diarrhoea and acute mesenteric ischaemia, once again pointing towards gastrointestinal risks of full EN [15].

Another recent RCT (EFFORT Protein trial) studied high-dose protein (≥ 2.2 g/kg/day) versus standard (≤ 1.2 g/kg/day) started within 96 h of ICU admission and continued for up to 28 days in mechanically ventilated patients. Results showed no difference in time to discharge alive from hospital, but possible harm from high protein in patients with baseline acute kidney injury and in the most severely ill [16]. A nested cohort sub-study of the EFFORT Protein trial in ventilated patients with shock showed that a difference in favour of early versus delayed EN was abolished after adjustment for severity of illness [17]. An observational study demonstrated, using a complex Cox-regression model, that moderate energy and protein delivery (10–20 kcal/kg/day and 0.8–1.2 kcal/kg/day) was associated with successful weaning and moderate energy delivery also with a lower risk of death [18].

### Interpretation of cumulative evidence

**Fig. 1:**
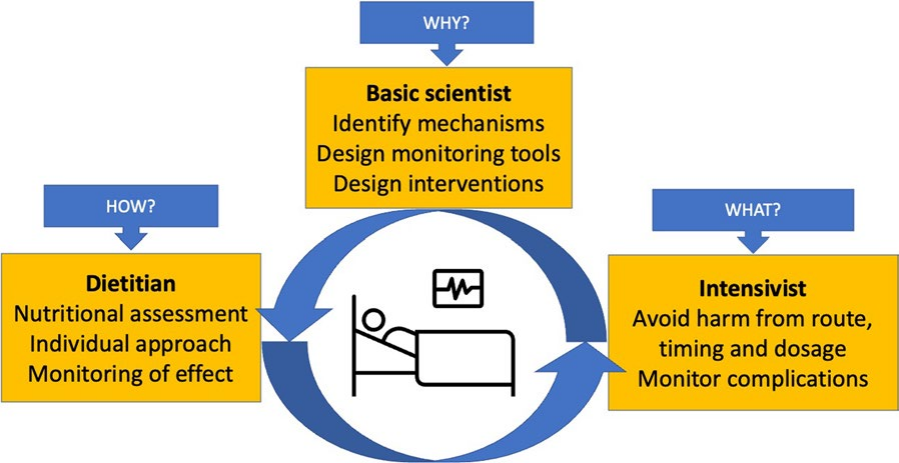
Different viewpoints with their main challenges to achieve optimal nutritional care of critically ill patients

In Fig. 1, we present three different viewpoints from a basic scientist, a dietitian and an intensivist that should complement each other to allow optimal nutritional care for critically ill patients with currently available evidence.

### Basic scientist’s viewpoint

Recent nutritional intervention trials (see above) suggest that early full nutrition is harmful in critically ill patients treated in an ICU on a group level and therefore current ESPEN guidelines recommend a slow start of nutrition. The clinical consequence of this is that most ICU patients nowadays receive less than full nutrition in the first few days up to a week of ICU treatment. This also has a potential downside since underfeeding [19] is related to a worse outcome, particularly in frail malnourished patients. From a basic scientist point of view, this means that we either are wrong about what ‘full nutrition’ for the patient is or there are biological mechanisms that lead to acute harm when giving critically ill patients otherwise adequate amount of energy and protein. Overfeeding in general leads to an increased risk of disease and early death, but this normally is a process that takes place over years to decades [20]. The intriguing question is why relative overfeeding during critical illness has an acute negative effect.

Determining a person’s nutritional needs is generally already a challenge and this becomes even more problematic when patients are critically ill, cannot eat themselves and need medical nutrition therapy. We have very few tools to determine the full nutritional needs of a patient. The only crude measure we have is measuring resting energy expenditure by indirect calorimetry [21]. This is much better to determine the individual patient’s energy expenditure than using equations [22], but it is a snapshot measurement with an analytical error that we use to guide the patient nutrition for a couple of days. Still, very few units have access to indirect calorimetry and use it frequently to guide nutrition clinically. Moreover, indirect calorimetry measurements often cannot be performed in the unstable patients. Most patients are unstable during the first days of ICU treatment when the risk for overfeeding is the highest and when we mostly would need a measurement of energy expenditure. So, we would benefit from better ways to assess energy expenditure in all patients when we need it, not when we can measure it.

But even when we know the patient’s energy expenditure, we cannot be sure that we should give the same amount of energy as is expended and how should we give this energy: as carbohydrates or lipids, or both. For energy, at least we have a tool to work with, but for estimating protein needs this is basically not available. In addition, proteins are mostly stored in functional tissue such as muscle, while energy is stored in adipose tissue with the sole purpose of storage. Loss of muscle protein, muscle mass and muscle function are causing major problems for critically ill patients especially during their recovery [23]. Despite this importance, we do not have tools to assess the individual patient’s protein needs. Moreover, post hoc analyses of both the PEPaNIC and EPaNIC trials [24, 25] suggest that protein overfeeding is mostly related to worse outcome.

Since we do not have the right tools to accurately and continuously determine the energy and protein needs of the individual patient, we generalise nutritional therapy on a group level. This means that many individual patients are underfed or overfed on different days. It seems that overfeeding leads to an acute disadvantage for the critically ill patients, and therefore, we assume that it is safest for all patients not to be overfed. With this approach, we are basically underfeeding many individual patients. The intriguing questions are why overfeeding is causing acute harm in the critically ill patient and whether this happens in the same way in all patients. Several mechanisms of harm have been proposed in the literature over the last decade, but all have rather weak evidence (see below). We need to better define the cellular or biological mechanisms of harm of overfeeding a critically ill patient. When we do understand these better, we will most likely be able to develop and validate new biomarkers for this signal of harm and use these to better feed the individual critically ill patient up to the real full nutrition (individual target) of that patient. The mechanisms that could potentially contribute to this harm and which are most commonly discussed are endogenous energy supply and autophagy [4–6].

It is thought that during the acute phase of critical illness a large part of the needed energy is supplied by the endogenous stores, and that this cannot be appropriately suppressed by exogenous nutrition. This leads to a relative overfeeding even when we think we are feeding according to the patient’s needs. This seems to be a plausible mechanism as several studies have shown that nutrition is unable to adequately suppress the de novo production of glucose (gluconeogenesis) in the critically ill [26, 27]. However, the same quality data is not available for the other parts of metabolism like the release of amino acids from protein degradation and fatty acids from lipolysis.

The role of autophagy was suggested from post hoc analyses of the EPaNIC study [28] and is based on the assumption that overnutrition will block autophagy and this will inhibit the breakdown of damaged intracellular proteins and organelles, which will prolong organ failure. The two main functions of autophagy are to degrade damaged protein complexes and to degrade nutritional reserves during fasting. Nutrition is therefore a strong signal for inhibiting autophagy. Several animal studies support this hypothesis [29]. On the other hand, it is difficult to measure the actual flux through the autophagy process in humans, and therefore, autophagy is often assessed with static markers. Serum from acutely critically ill patients is able to block the autophagy flux in incubated human muscle cells. However, serum from only about 15% of the patients blocked autophagy flux, while about 15% stimulated autophagy flux [30]. These responses were mainly related to the severity of the patient’s disease rather than to nutritional therapy. These results indicate that the role of autophagy, and its regulation by nutrition is complex. However, this also means that we can use these individual responses to learn more about the regulation of cellular processes due to disease and the possibility to modify by nutrition. Hopefully in future, this leads to novel biomarkers to guide nutritional therapy for the individual patient.

Besides these mostly discussed mechanisms, there are other possible acutely detrimental cellular effects of nutrients that should be investigated in more detail. The toxic effects of lipid metabolites such as ceramides [31] or the direct cellular toxic effects of too much glucose [32], but also the beneficial effects of the ability to stimulate the production of ketone bodies [33], all could play a role in the complexity of critical illness and nutritional needs.

Overall, for every individual critically ill patient there probably is an optimal nutritional target, which probably changes over time and which we currently cannot determine. However, there are good clinical trials showing that too much can be acutely harmful, and there are many proposed mechanisms for this signal of harm. We just need to explore these better to find the real mechanism of harm, and from this, develop new biomarkers that can hopefully be used in the future to truly individualise nutrition in the ICU.

### Dietitian’s viewpoint

The presence of specialist ICU dietitians is not ubiquitous across the world. However, where they do exist, they play a key role in nutrition assessment (e.g. diagnosing malnutrition), individualising nutrition targets and monitoring for signs of both underfeeding and overfeeding with adjustments made to feeding targets based on their thorough assessment. It is clear from recent studies that the role of the ICU dietitian is more important than ever with the focus shifting from the ‘one size fits all’ feeding approach to one where individualisation may be the key factor in driving positive outcomes for patients. When determining individualised nutrition interventions for critically ill patients, the dietitian will consider the patient population, the individual patient’s nutritional status and the expected outcome and formulate an appropriate feeding regimen based on this. From a dietitian’s perspective, there are several key points that stand out in the recent trials mentioned above.

First, both NUTRIREA-3 and FRANS support the current ESPEN guideline recommendation for hypocaloric feeding early during critical illness to account for the endogenous production of glucose which, to date, cannot be measured at the bedside. Coupled with an appropriate enteral feeding protocol, hypocaloric feeding typically occurs naturally as a function of critical illness given the first few days of ICU admission are characterised by feeding interruptions for investigations, procedures and GI intolerance. The results of FRANS and NUTRIREA-3 come as no surprise given that target energy was delivered from day one in NUTRIREA-3 (25 kcal/kg) and around 90% of target energy was met on day two in FRANS. It remains unknown when a patient shifts from the acute phase to the recovery phase and none of the recent trials provide any further insights into this with study protocols being based on day of ICU admission rather than any objective measure. Clearly, just counting ICU days is an inappropriate way to determine the transition to the recovery phase. This is a major barrier to the nutrition care of ICU patients, but until further studies are available on this topic, it seems reasonable to aim for an individualised approach from day 3–5 of ICU admission once the patient is haemodynamically stable, and there may be a clearer idea of the trajectory of care.

Second, the patient population is an important consideration when interpreting the results of these recent trials. Seemingly lost in the discussion around NUTRIREA-3 is that the patient population included those who were in shock and receiving high dose vasopressors (median 0.5 ug/kg/min in both groups) although the definition of shock is not clear. It is unusual practice to aim for 100% of the energy target in early critical illness, especially for those receiving high dose vasopressors and it is clear that this practice should continue to be avoided. Similarly, there were sub-groups of patients in the EFFORT protein trial who had worse outcome when high dose protein was delivered (those with baseline AKI and high baseline SOFA scores). The harm associated with high doses of energy and protein in particular sub-groups of patients certainly highlights the importance of a more individualised approach to feeding the critically ill patient and one could argue that the ICU dietitian is best placed to manage this given that a feeding protocol cannot account for every patient eventuality. A more individualised approach would also allow alternative feeding strategies for those who are malnourished or at risk of refeeding syndrome.

Finally, interpreting the outcome of any clinical trial in terms of biological plausibility and patient consideration is essential. Recent years have seen a shift towards exploring the relationship between nutrition delivery and muscle mass, physical function and quality of life rather than a sole focus on mortality. NUTRIREA-3 found a 1-day difference in the outcome of ‘readiness to ICU discharge’, but it is unclear whether this difference translated into meaningful improvements in physical function or quality of life in the months following ICU discharge as these measures are not reported. However, the MRC-Sum score was 3-points higher at ICU discharge in the patients receiving full nutritional targets although this was not statistically different, and the study was not powered to determine this. Whether a one day longer ICU stay can be considered ‘harm’ in this patient group is highly debatable. Indeed, no length of stay outcome was included in the core outcome set for trials of nutrition and metabolism [34], and patients have previously expressed fear and anxiety at stepping down from ICU to the ward [35]. Undoubtably, there is a cost implication involved, but without knowing the longer-term impact, it is impossible to interpret this outcome, particularly in light of an unblinded trial. A similar question remains around the EFFORT Protein Trial where the effect of the higher dose of protein on muscle mass and physical functional recovery remains unknown, but sub-studies are underway exploring this outcome for which the results will be welcome.

Overall, recent trials of nutrition in the critically ill provide strength to the current ESPEN guidelines around the early dose of energy and protein and give weight to the argument for individualised nutrition assessment and feeding regimens. However, strategies to effectively define the shift from the acute to recovery phase are needed.

### Intensivist’s viewpoint

Findings of the most recent studies may easily be (mis)interpreted as ‘do not feed early’ or ‘do not increase dose from the minimal during the first week in the ICU’. However, this is probably not a correct interpretation and would put the pendulum towards underfeeding of all ICU patients once again, instead of searching for the optimum.

We have learnt that in the early period, almost all critically ill patients (independent of their body weight and character of illness, etc.) may be handled in a similar way with administering low amount of all macronutrients. Accordingly, all differences between malnourished and well-nourished patients are abolished in nutritional management during the acute period, making it easy for a clinician usually focused on circulatory and respiratory problems. Indeed, low dose and slowly increased feeding not only avoids harm due to overfeeding in state of increased endogenous energy production, and via suppression of autophagy, but also avoids refeeding syndrome. Earlier concepts of reaching energy targets faster in malnourished patients in fact carried a considerable risk of triggering refeeding syndrome in these patients. This might have often gone unnoticed due to rarely measuring serum phosphate levels [36–38], which dynamics are considered as a marker for refeeding syndrome [39].

At the same time, it may be dangerous to continue such an easy ‘one (small) size fits all’ approach for prolonged time periods. Even though not directly visible, focus on nutrition in the ICU and beyond has probably contributed to overall improving outcomes. Clearly, many patients might have been harmed with early overfeeding and refeeding, as well as serious GI complications with EN, but at the same time many might have profited from avoidance of underfeeding. Therefore, we should be aware of the risk of interpreting available studies as a proof for benefit of delaying nutrition or of prolonged underfeeding. Instead, all these studies just confirm the harm from early full feeding.

We have to admit that it is currently unclear at which time point, in which dose and which route to start nutrition, and when and how to increase the feeding rate. Accordingly, we just aim to avoid harm without knowing how to make patients benefit from nutrition.

A rationale to start nutrition early (during the first 48 h) is an anticipated delay in reaching energy targets with slow progression of feeding rates. The concept of Nutrirea-3 to keep 6 versus 25 kcal/kg/day for the first 7 days, was most likely appropriate for this study, but should not be taken over to clinical practice. Administering all patients 6 kcal/kg/day for 7 days and thereafter going promptly to 30 kcal/kg/day may carry a very high risk of refeeding syndrome. It is especially dangerous, when such abrupt transition from low to high dose coincides with a transfer from the ICU to the normal ward with less capability to detect refeeding syndrome. Slow progression of nutrition dose has a physiological rationale, whereas ‘slow’ is not defined.

There is rather a strong physiological rationale to prefer enteral route over parenteral with feeding the mucosa and the microbiome and possibly maintain signalling mechanisms [40]. However, GI complications of EN in patients receiving vasopressors are of concern. Although differing between units, considerable proportion of patients admitted in the ICUs receives vasopressors [41].

Whether early EN at a low rate with a slow progression may be beneficial compared to early high dose EN, early low dose PN and delayed nutrition via any route has not been proven in studies. Importantly, patient preferences regarding EN versus PN have not been considered and are likely to be different from healthcare professionals.

Early start and slow progression of EN may improve tolerance of enteral feeding [42, 43]. At the same time, it is unclear whether and when enteral feeding intolerance should be managed with prokinetics or postpyloric feeding and when it could be just observed with keeping the rate of EN (very) low. Studies on harm of full EN support the hypothesis that enteral feeding intolerance could be an adaptive mechanism aiming to limit the energy intake in the early period of critical illness. Accordingly, enteral feeding intolerance should probably not be aggressively treated in this period.

## Translation of evidence into practice (how to avoid harm)

**Fig. 2:**
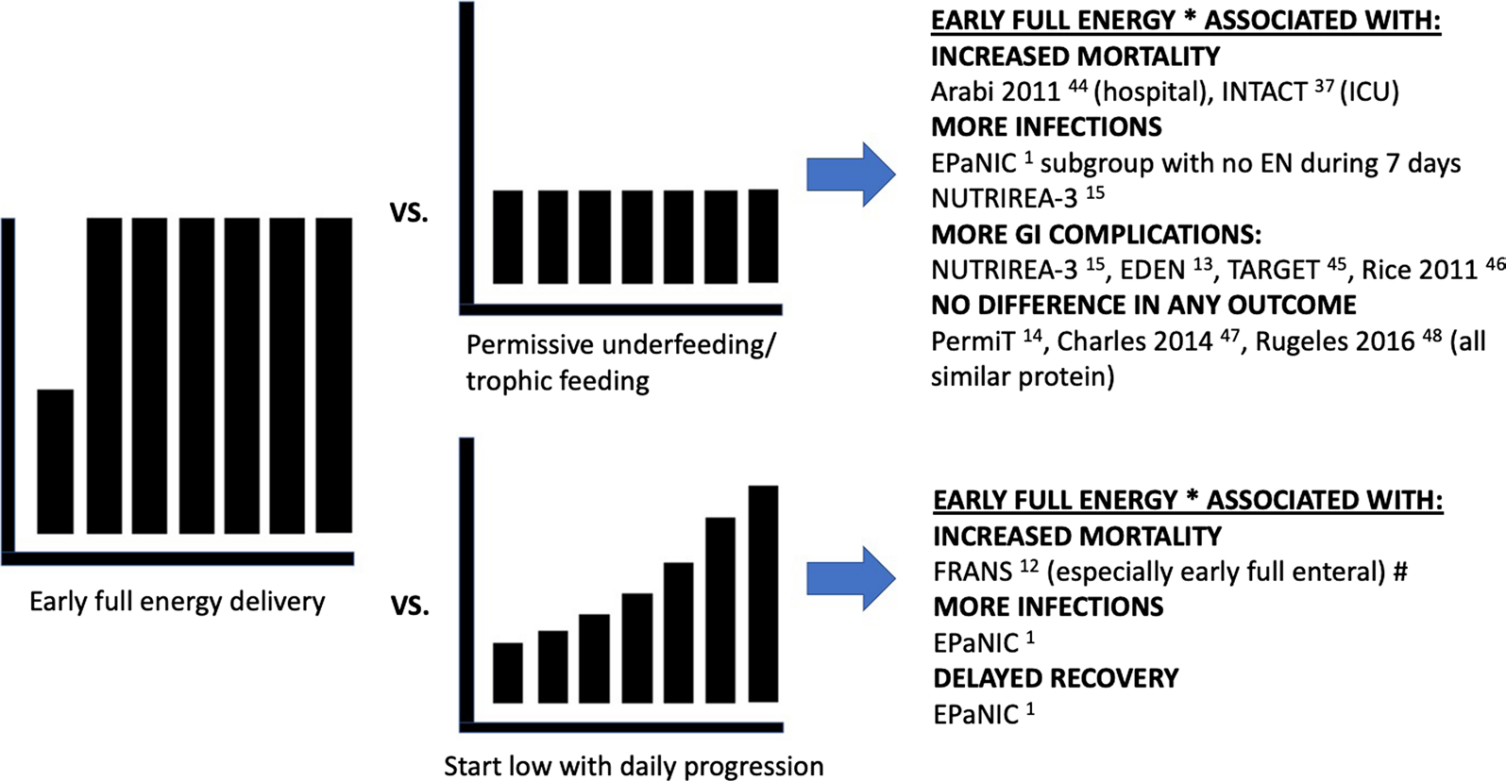
Summary of evidence on early full feeding versus other strategies. The graphs here are schematic, summarising the approaches in general, whereas the exact timing, dose, slope and duration of intervention differ between studies. *Any route, non-nutritional calories included where available. # Non-RCT, propensity score for analysis

Evidence on early full feeding versus other strategies is summarised in Fig. 2 [1, 12–15, 37, 44–48].

**Fig. 3:**
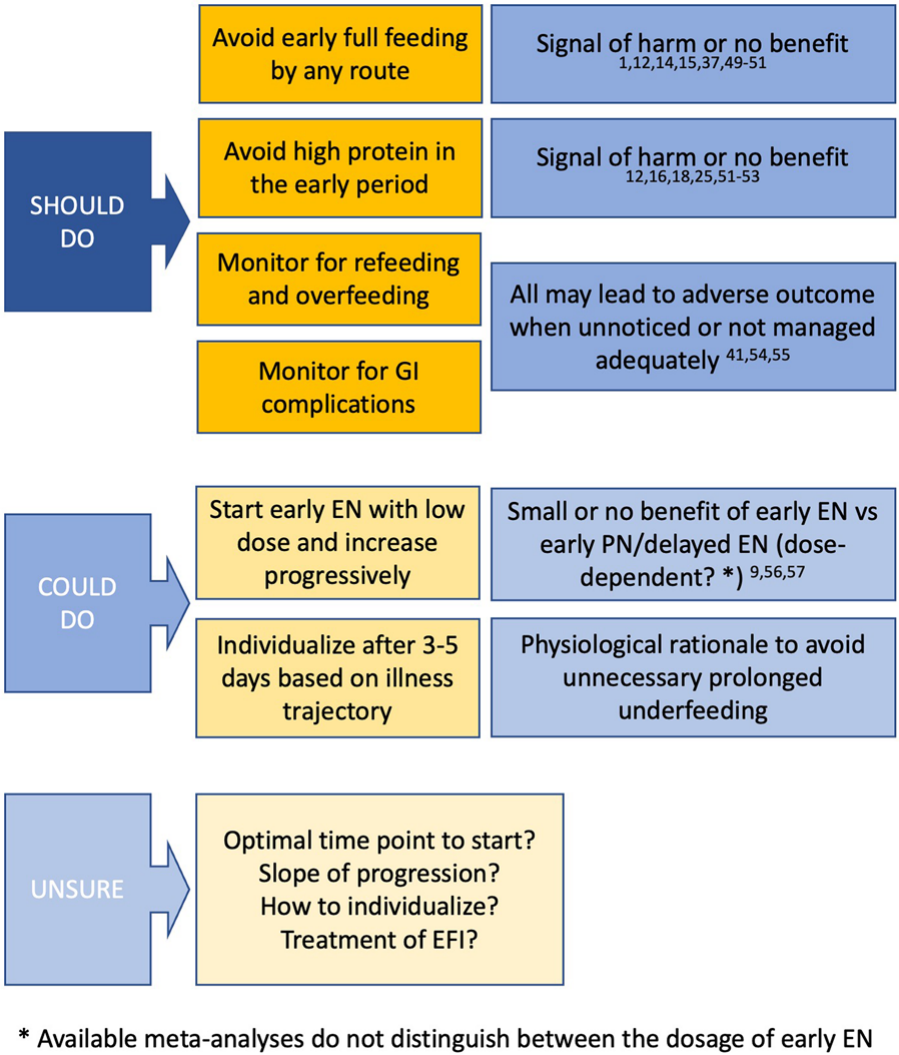
Practical summary of current understanding on nutrition. EFI—enteral feeding intolerance; EN—enteral nutrition; GI—gastrointestinal

Practical summary of current knowledge and uncertainties is shown in Fig. 3 [1, 9, 12, 14–16, 18, 25, 37, 41, 49–57].

### What do we know one should not do

- Do not provide full (70–100%) energy target in the first few days of admission.
- Do not provide high dose protein in the first few days of admission.

### What do we think one should do

- Start nutrition within the first few days of admission.
- Start with a low dose of EN if not contraindicated. If contraindicated, start with a low dose PN (consider also non-nutritional calories).
- Use indirect calorimetry after day 3 to reduce the risk of overfeeding the individual patient.
- Monitor for GI dysfunction to avoid or early identify severe complications of EN.
- Not treat enteral feeding intolerance aggressively just to maximise the amount of EN during the first days of admission.
- Monitor metabolic response (e.g. insulin requirements) to feeding.
- Monitor for refeeding.
- Progress slowly with energy and protein concomitantly, independent of the route.
- Avoid prolonged underfeeding with individualisation of nutrition targets from day 3–5 after ICU admission. Number of days is arbitrary. Daily clinical evaluation of disease trajectory to estimate the time point of transition to recovery phase is warranted despite the absence of precise markers.

### What do we still need to find out

- Is early EN at a low rate with a slow progression beneficial compared to early high dose EN? However, the evidence on harm from early full EN evokes ethical concerns about this study objective.
- Is early EN at a low rate with a slow progression beneficial compared to low dose PN and delayed nutrition via any route?
- Is there any specific subgroup of critically ill patients who might profit from early full feeding by any route?
- Which clinical outcomes can best describe the patient's response to the level of feeding in studies?
- Which nutritional outcomes are most important from patient perspective?
- Identify biomarkers reflecting shift from acute to recovery phase.
- If one has a second (or third etc.) hit of sepsis throughout the ICU stay, should we manage it in a similar way as the first hit?
- If targeting protein to lean mass rather than actual body weight improves outcome?
- Develop better bedside tools and biomarkers for monitoring nutritional needs for the individual patient.
- Find the true biological or cellular mechanisms of harm from overfeeding and develop novel biomarkers from this.
- Better define a refeeding syndrome specific for the critically ill patient.
- Continue validating and refining tools for assessment of GI function.

## Conclusions

Cumulative evidence indicating no benefit of early full nutrition has been expanded by recent studies. Administering early full nutrition by any route and high amounts of protein may harm patients and should not be practiced based on current knowledge. However, next to stating what one should not do, there is much more uncertainty regarding what one should do, e.g. when to start via which route and how to determine an optimal target for an individual patient. Basic research is needed to further explore the mechanisms of harm from early full nutrition and to identify tools to monitor metabolism, thereby assisting in design of clinical studies moving from the group approach to a more individual approach.

## Data Availability

Not applicable.

## Abbreviations

EN: Enteral nutrition
ESPEN: European Society for Clinical Nutrition and Metabolism
ICU: Intensive care unit
PN: Parenteral nutrition
RCT: Randomised controlled trial
